# Adipokines in the Skin and in Dermatological Diseases

**DOI:** 10.3390/ijms21239048

**Published:** 2020-11-28

**Authors:** Dóra Kovács, Fruzsina Fazekas, Attila Oláh, Dániel Törőcsik

**Affiliations:** 1 Department of Dermatology, Faculty of Medicine, University of Debrecen, Nagyerdei krt. 98., 4032 Debrecen, Hungary; kovacs.dora@med.unideb.hu (D.K.); fruzsi.fazekas28@gmail.com (F.F.); 2 Department of Physiology, Faculty of Medicine, University of Debrecen, Nagyerdei krt. 98., 4032 Debrecen, Hungary; olah.attila@med.unideb.hu

**Keywords:** adipokines, keratinocytes, fibroblasts, sebocytes, melanocytes, hair growth, psoriasis, atopic dermatitis, acne, melanoma

## Abstract

Adipokines are the primary mediators of adipose tissue-induced and regulated systemic inflammatory diseases; however, recent findings revealed that serum levels of various adipokines correlate also with the onset and the severity of dermatological diseases. Importantly, further data confirmed that the skin serves not only as a target for adipokine signaling, but may serve as a source too. In this review, we aim to provide a complex overview on how adipokines may integrate into the (patho) physiological conditions of the skin by introducing the cell types, such as keratinocytes, fibroblasts, and sebocytes, which are known to produce adipokines as well as the signals that target them. Moreover, we discuss data from in vivo and in vitro murine and human studies as well as genetic data on how adipokines may contribute to various aspects of the homeostasis of the skin, e.g., melanogenesis, hair growth, or wound healing, just as to the pathogenesis of dermatological diseases such as psoriasis, atopic dermatitis, acne, rosacea, and melanoma.

## 1. Introduction

White adipose tissue (WAT) is present throughout the human body with a pivotal role in lipid storage, thermoregulation, mechanical organ protection and regulation of energy homeostasis [1], but with remarkable anatomical, ontogenic, metabolic, cellular, molecular and physiological differences between its two forms, namely the visceral and the subcutaneous ones [2,3]. Importantly, WAT is also an active endocrine tissue due to the secretion of various hormones and bioactive molecules called adipokines [4], and has a central role in the regulation of immune responses locally and systemically; thus, it is considered as an immunometabolic system [5]. Alterations in the number of WAT cells and their dysregulated functions, therefore results in the pathogenesis of various diseases such as obesity, insulin resistance, type 2 diabetes, cardiovascular diseases [6,7] but may also be involved in “non-typical metabolic diseases” such as psoriasis [8,9].

Adipokines are small molecular weight biologically active proteins primarily produced by adipocytes, however many members are expressed and secreted also by non-adipocyte cells [10]. As opposed to protein families—which share a specific domain structure and homologous, conserved amino acid sequence—adipokines constitute a merely functional category of diverse proteins and peptides which are active in cell signaling. Interestingly, beyond the significant conformational and structural differences across the adipokines, different isoforms may exist even for individual adipokines (e.g., adiponectin or apelin) [11]. Furthermore, adipokines (such as adiponectin, the member of the serine protease inhibitor (SERPIN) gene family (SERPINE1) or apelin) may undergo further structural reorganization when involved in pathological process [11,12].

Not surprisingly, their receptors have a diverse repertoire as well, accounting for the wide range of signaling pathways which may be activated by adipokines. While some of the adipokines transmit their signals through designated receptors, others act through receptors of various cytokines and hormones [12]—for example, leptin exerts its biological effect through the leptin receptor (Ob-R), which belongs to the type I cytokine receptor family [13]; the receptors for chemerin, monocyte chemoattractant protein-1 (MCP-1) and apelin are G protein-coupled receptors (GPRs) [14,15,16], while effects of resistin, visfatin and fetuin-A are mediated through the insulin receptor and a pattern recognition receptor (Toll-like receptor [TLR] 4) [17,18,19]. Adiponectin receptors are the members of the progestin and adipoQ receptor family, while the zinc-α2-glycoprotein (ZAG) is a β2 adrenergic receptor agonist [20,21]. SERPINE1 binds to the urokinase receptor, lipocalin 2 (LCN2) is able to act through its own specific 24p3 receptor and in the case of omentin-1, no specific receptor has been identified so far [22,23,24]. Moreover, multiple isoforms of a given receptor could be present as well on various cell types, as it is in the case of Ob-R, which has multiple splice variants—but only the long isoform (Ob-Rb) contains all the intracellular motifs that are necessary to activate the Janus kinase 2/signal transducer and activator of the transcription factor 3 (JAK2/STAT3) pathway [25].

Currently, more than 600 adipokines have been described in the human body with various biological properties. The most widely used classification places their inflammatory properties in the center and distinguishes pro-and anti-inflammatory adipokines. Up-regulation of pro-inflammatory adipokines leads to the development of a chronic, low-grade inflammatory state and contributes to metabolic dysfunction. On the other hand, the adipose tissue secretes certain anti-inflammatory adipokines as well, which are under intensive investigation regarding their role in these processes [9,26]. Although adipokines are the key mediators of various adipose tissue-induced and -regulated inflammatory diseases, it has become evident that they may also be active regulators of cutaneous inflammatory processes, which promoted the identification of adipokine source and target cells in the skin [27,28,29]. Up to now, adipokine research has acquired an indisputable place in dermatology, with a growing body of information regarding the role of adipokines in skin (patho) physiology. In this review, with the limitation of not involving interleukins and the skin-associated immune cells, we summarize the adipokines produced by different human skin cell types, and demonstrate how these adipokines may contribute to the development of various skin diseases.

## 2. Expression of Adipokines in Skin Cells

### 2.1. Keratinocytes

**Figure 1:**
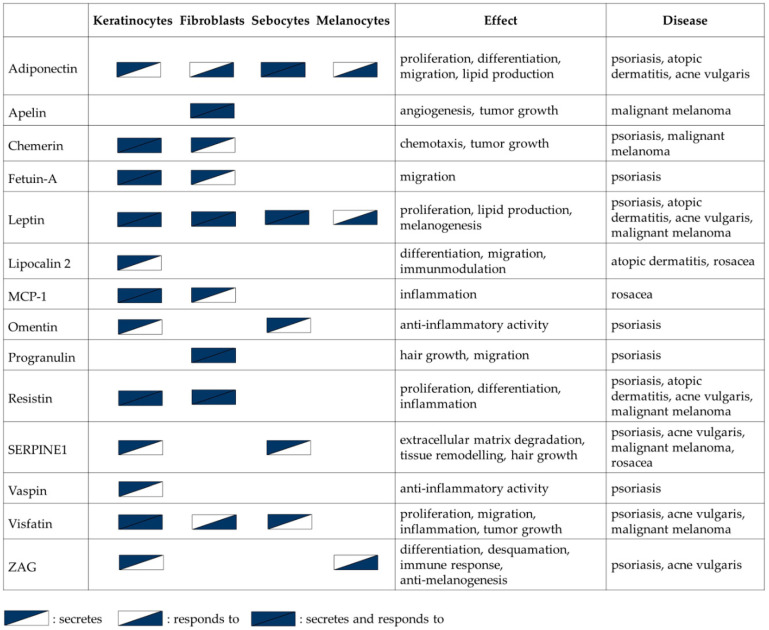
Overview of adipokines expressed and secreted by different human skin cell types and their effects on these cells.

Continuously proliferating and differentiating keratinocytes are the dominant cell type of the epidermis. Keratinocytes are able to secrete and to respond to a wide range of bioactive molecules including adipokines, thereby regulating skin homeostasis (Figure 1).

Leptin, encoded by the “obese” (Ob) gene [30], is one of the first discovered and most-studied adipokines in skin biology. Generally, leptin acts as a regulatory factor through the central nervous system, balancing food intake and energy expenditure [31]. In addition to the JAK2/STAT3 signaling, Ob-Rb is able to regulate members of the mitogen-activated protein kinase (MAPK) family (p38 MAPK), as well as, the stress activated c-Jun N-terminal kinase (JNK) and phosphatidylinositol 3-kinase/protein kinase B (PI3K/Akt) pathways [25,32,33]. The common endpoint of these signaling pathways is nuclear factor kappa-B (NF-κB), the major transcription regulator of inflammatory mediators. Therefore, it is generally accepted that in the human skin, leptin acts as a pro-inflammatory adipokine [34]. In the human epidermis, prominent leptin immunoreactivity could be observed in the basal and suprabasal layers and its biologically active receptor (Ob-Rb) was also found to be expressed by keratinocytes [35]. The leptin/STAT3 pathway is linked to the differentiation, proliferation and migration of keratinocytes and it is a potent regulatory factor of innate and adaptive immunity. In human keratinocytes in vitro, leptin is able to stimulate the expression of the antimicrobial peptide human β-defensin-2 (hBD-2) as well as the production of pro-inflammatory cytokines (interleukin [IL]-6, IL-8 and tumor necrosis factor-α [TNF-α]) via STAT1, STAT3, JAK2, p38 MAPK and NF-κB pathways. [36,37]. However, in transgenic mice overexpressing leptin in keratinocytes, although high serum leptin levels could be detected, a normal histological morphology of the epidermis and the epidermal appendages were observed with unaltered expression of differentiation and proliferation markers [38]. Whether these findings are mice specific, or highlight the importance of investigating the role of leptin in combination with other factors, calls for further studies.

Visfatin (also known as pre-B-cell colony-enhancing factor (PBEF), or nicotinamide phosphoribosyltransferase (NAMPT)) was originally identified in human peripheral blood lymphocytes and was found to enhance the maturation of B-cell precursors [39]. In human keratinocytes, the expression of visfatin could be induced by epidermal growth factor (EGF) treatment and was able to enhance the proliferation and migration of these cells via extracellular signal-regulated kinases (ERK) 1/2 and JNK1/2 pathways [40]. In vitro experiments showed that visfatin activated the NF-κB, STAT3 and CCAAT/enhancer-binding protein (C/EBP) pathways, increasing the basal and TNF-α-induced mRNA expression and secretion of chemokines (CXCL8, CXCL10) and antimicrobial peptides (cathelicidin, hBD2, hBD3) in normal human keratinocytes (NHKs) [41,42]. Although it is obvious that visfatin acts as a pro-inflammatory mediator under the conditions studied so far [43,44], further investigations are needed to understand its role in the immunobiology of keratinocytes.

Recent in vitro evidence showed that LCN2 (also known as neutrophil gelatinase–associated lipocalin (NGAL)) is another potential immunomodulatory adipokine as it was able to induce immune tolerance through modulation of regulatory T cell expansion [45]. Although both normal hair follicles and in vitro cultured keratinocytes express LCN2, its strong induction in the epidermis can only be observed in certain skin diseases suggesting an association with pathological conditions in human skin [46]. In line with this, IL-1β, IL-17 and TNF-α treatment stimulated the secretion of LCN2 in NHK and HaCaT keratinocytes, and by ERK1/2 and p38 MAPK pathways, LCN2 was found to induce neutrophil chemotaxis [47,48]. Interestingly, a characteristic high expression of LCN2 was detected in the apical layer of the epidermis in sebaceous gland (SG) rich human skin as opposed to SG poor areas suggesting that LCN2 (together with other cytokines and antimicrobial peptides) has a role in the maintenance of the specific immune milieu of SG rich skin [49].

SERPINE1 (or plasminogen activator inhibitor type-1 (PAI-1)) has a major role in the modulation of extracellular matrix degradation and tissue remodeling [50]. The mRNA and protein expression of SERPINE1 are undetectable in normal mouse epidermis. However, its strong expression could be observed after mechanical injury or topical treatment with a specific NF-κB activator [51,52].

Adipokines with chemotactic properties are also found in keratinocytes. Chemerin (also known as retinoic acid receptor responder 2 (RARRES2), or tazarotene-induced gene 2 protein (TIG2)), has been introduced as a chemokine as it is able to exert a chemotactic effect through the p38 MAPK pathway on dendritic cells (DCs) and natural killer cells (NK cells) and regulates macrophage-mediated immune responses [53]. The mRNA and protein expression of chemerin and its receptors (chemerin chemokine-like receptor 1 (CMKLR1) and (GPR1)) show a characteristic distribution in human skin. While chemerin is expressed primarily in the epidermis, CMKLR1 and GPR1 expression tends to be higher in the dermal compartment [14]. The dermal response to chemerin is most probably a result of the release from its site of synthesis in the epidermis triggered by danger signals. In human skin cultures as well as in mouse skin in vivo, microbial stimuli increase chemerin synthesis indicating that the epidermis is a functional microbe-responsive site of chemerin production [14]. In the same experimental models, psoriasis-related cytokines, IL-17 and IL-22 significantly down-regulated chemerin expression [14]. Based on in vitro experiments, chemerin facilitated the secretion of IL-1β, IL-8, TNF-α, as well as of reactive oxygen species (ROS), and consequently activated the NF-κB signaling in NHK and HaCaT cells [54]. Overexpression of bioactive chemerin by basal keratinocytes in transgenic mice revealed that chemerin is able to significantly delay tumor development and progression in a DMBA/TPA model of skin carcinogenesis [55].

In addition to chemerin, MCP-1, also known as C-C motif ligand 2 (CCL2) also has chemotactic activity with a primary function to recruit immune cells (e.g., monocytes, memory T lymphocytes and NK cells) to the site of inflammation [56]. In human keratinocytes, via p38 MAPK and NF-κB pathways, chemical stimuli or inflammatory factors (e.g., TNF-α and interferon-γ (IFN-γ)) are able to induce the mRNA and protein expression/secretion of MCP-1 but its presence is not detectable under basal conditions [57,58,59]. In vitro experiments have shown that MCP-1 treatment increased p53 and p21 protein expression in NHKs through p38 MAPK signaling, suggesting that it might be involved in keratinocyte senescence [60]. Further animal studies proved that subcutaneous injection or epidermis-specific expression of MCP-1 induced neither spontaneous cutaneous inflammation nor did it result in tissue damage, but stimulated the recruitment of DCs and Langerhans cells into the epidermis [61,62]. Thus, it can be speculated that MCP-1 alone is inefficient to induce a local inflammatory response.

Resistin is a cysteine-rich protein that influences glucose homeostasis through the regulation of the suppressor of cytokine signaling 3 (SOCS-3) [63]. With low levels of expression, resistin can be detected in the cytoplasm of the outer root sheath and epidermal keratinocytes [64], however, little is known about its effect on these cells, as monocytes and macrophages appear to be its major source and target cells [65].

Retinol-binding protein (RBP) 4 mobilizes retinol (vitamin A) from the liver to target tissues where it is taken up through the transmembrane protein stimulated by retinoic acid 6 (STRA6) [66]. Although STRA6 is constitutively expressed in NHK and HaCaT keratinocytes and down-regulation of this receptor leads to hyperproliferation in vitro and in vivo skin models [67], the role of RBP4 in human keratinocytes is not fully known and clarified.

Apelin is an endogenous ligand of the GPR called APJ [16]. Although the expression of apelin in the epidermis has not been studied, transgenic overexpression of apelin in mouse keratinocytes resulted in enlarged blood vessels in the dermis which was mainly mediated by endothelial cell aggregation [68]. This is not surprising because APJ is structurally related to the angiotensin II receptor type 1, and one of its major functions is the regulation of angiogenesis [69].

Fetuin-A can be produced primarily by hepatocytes and adipocytes, but keratinocytes and fibroblasts were also proposed to secrete it [70,71]. Low levels of fetuin-A was detected in the epidermis and in primary human keratinocytes, mainly at the cell membrane and in the cytoplasm [71]. Although fetuin-A results in morphological changes in HaCaT and primary keratinocytes and significantly promotes the migration of these cells in an EGF receptor (EGFR)-independent manner, further studies are needed to clarify its role in the homeostasis of keratinocytes.

ZAG is involved in the regulation of obesity-associated inflammatory responses in hepatocytes [72]. Normal keratinocytes express ZAG which plays an important role in the terminal differentiation of these cells and thus in the cohesion of the stratum corneum [73].

Besides pro-inflammatory adipokines, anti-inflammatory adipokines are also found in the epidermis, although in smaller amounts. Adiponectin (ACRP, AdipoQ) is predominantly involved in the regulation of insulin sensitivity and whole body energy metabolism through its cell surface receptors (ADIPOR1 and 2) and via the AMP-activated protein kinase (AMPK) pathway [74]. Based on experiments performed in mouse models, disruption of both receptors abrogated adiponectin binding and action, but the two receptors affected different pathways with opposing effects [75]. As an anti-inflammatory adipokine, adiponectin is able to inhibit TLR-mediated NF-κB signaling, thereby abrogating lipopolysaccharide (LPS) induced TNF production and stimulating the release of IL-10 and IL-1 receptor antagonists (IL-1Ra) from human monocytes/macrophages [76,77,78]. In the human epidermis, adiponectin receptors are expressed mainly in the basal cell layer [79]. In NHK and HaCaT keratinocytes adiponectin is capable of enhancing cell proliferation, differentiation and migration, of increasing lipid contents via Sirtuin 1, and of inhibiting TNF-α and IL-6 secretion [80,81,82]. Furthermore, adiponectin down-regulates ultraviolet B (UVB) induced mRNA expression of hBD2 through the suppression of p38 and JNK MAPK signaling [83] and also acts on NHK filaggrin synthesis indicating that it may also be involved in skin diseases associated with disrupted barrier functions [84].

Omentin is also present in the epidermis of normal human skin, and its decreased protein expression has been described under pathological conditions, suggesting that omentin has an anti-inflammatory effect [85]. The evaluation of the role of omentin-1 with relevance to keratinocyte function requires further experiments.

The serpin protease inhibitor vaspin (SERPINA12) is also considered to be an anti-inflammatory adipokine. Keratinocytes are the major vaspin-expressing cells in the human skin [86]. Higher vaspin levels could be measured in differentiated keratinocytes, suggesting that its expression is associated with the maturation of these cells [87]. In co-cultures with immune cells, vaspin was able to modulate the crosstalk between keratinocytes and immune cells and attenuated the production of cytokines and chemokines of the inflammatory cells [87].

### 2.2. Fibroblasts

The most abundant cell types in the dermis are fibroblasts, which are the major source of extracellular matrix components (collagen, elastin, and fibronectin), thus playing a fundamental role in developing the proper structure and elasticity of the skin. Given the close ontogenic relationship between fibroblasts and adipocytes, it is not surprising that fibroblasts are also able to produce adipokines [88,89] (Figure 1).

mRNA and protein expression of leptin and its full-length receptor can be observed in human skin in vivo and in cultured fibroblasts [35,90]. In addition, leptin production is also detectable under basal conditions and can be induced by various stimuli [90,91]. Based on in vitro experiments, leptin has no effect on cell proliferation; however, it up-regulates hyaluronic acid production, reduces UV-induced TNF-α release and matrix metalloproteinase (MMP) expression [92].

Although adiponectin is not expressed at mRNA level in human dermal fibroblasts, its receptors are present in these cells and are up-regulated after UV irradiation and wounding suggesting that fibroblasts are potential adiponectin-responsive cells in the dermis [80,93,94]. In extracellular matrix regulation, adiponectin may exert a potent anti-fibrotic effect via AMPK signaling, while its impaired production is accompanied by abnormal fibroblast activation [95]. Moreover, exogenous adiponectin administration down-regulated the MMP expression and prevented UV-induced dermal matrix degradation via suppression of EGFR signaling in human foreskin fibroblasts [94].

Regarding chemoattractant proteins, although relatively high chemerin receptor expression can be observed in the dermis, chemerin itself is hardly detectable under normal conditions [14]. Its transcription and protein expression is however increased in senescent human dermal fibroblasts, enhancing the chemotaxis of tumor cells via activation of MAPK signaling [96]. Dermal fibroblasts also produce and secrete MCP-1, which is positively regulated by growth factors in both an auto- and a paracrine manner [15].

mRNA and protein expressions of apelin are also detectable in normal human fibroblasts, which can be reduced by transforming growth factor β (TGFβ) 1 stimulation. Vice versa, TGFβ1-induced fibrosis-related gene expression was inhibited by apelin treatment suggesting that apelin might have a potential role in blocking TGFβ1/Smad signaling under pathological conditions in dermal fibroblast [97].

High levels of fetuin-A were found throughout the dermal layers, with fibroblasts stained positive for fetuin-A; however, further details are needed to conclude on any of its possible biological relevance [71].

Progranulin was not detected at basal conditions in fibroblasts but its mRNA levels increased after injury [98].

### 2.3. Sebocytes

SGs together with the hair follicle (HF) form the pilosebaceous unit with a primary role to produce sebum to cover the hair and the skin [99]. However, studies confirmed that the role of sebocytes is not limited to the production of lipids, but may have inflammatory roles as well in which sebocytes may utilize adipokines [99,100,101] (Figure 1).

Intense leptin and leptin receptor expression was observed in the SGs under normal as well as pathological conditions [101,102]. Moreover, sebocytes were capable of increasing the secretion of leptin when activated with TLR2 and 4 activators [101]. Importantly, in response to leptin, sebocytes exerted pro-inflammatory responses such as enhanced inflammatory enzyme (5-lipoxygenase (5-LOX) and cyclooxygenase-2 (COX-2)) and cytokine production (IL-6 and IL-8) via the activation of the NF-κB and STAT3 pathways, in vitro [102]. Based on further in vitro and in vivo experiments, leptin is involved in a complex regulation of lipid production in mice sebocytes by inducing the diglyceride acyltransferase (DGAT)-regulated sebaceous lipogenesis, promoting the enlargement of the intracellular lipid bodies and increasing the ratios of the produced unsaturated/saturated fatty acids [102,103]. Activated mammalian target of rapamycin complex 1 (mTORC1) signaling, a master regulator of sebocyte differentiation, also enhances sebocyte-derived leptin production, while leptin further stimulates mTORC1 and sterol regulatory element binding protein 1 (SREBP1) expression, which may have an important role in the pathogenesis of SG-associated diseases [104].

Although immunohistochemical analysis revealed that in vivo SGs and in vitro SZ95 cells expressed resistin, visfatin, SERPINE1 and omentin-1, the role of these adipokines in the biology of SGs is not fully elucidated [64,85,101]. Resistin was predominantly localized in the basal cells of SGs—suggesting that it may play a role in the regulation of sebocyte proliferation and differentiation [64] —but with further studies needed to determine its role in SG physiology.

The high levels of visfatin and SERPINE1 in sebocytes, which were also further increased upon TLR stimulation, support that these adipokines could be involved in the pro-inflammatory potential of SGs and may contribute to a specific dermal milieu and to extracellular matrix remodeling under pathological conditions [101].

Adiponectin and its receptors are also expressed in SGs of normal human skin and primary sebocytes [79,101,105]. In addition to promoting proliferation, adiponectin efficiently activates the AMPK/Akt pathways, induces the expression of SREBP1 and significantly up-regulates the production of lipids such as cholesterols, triglycerides, wax esters and squalene in primary sebocytes [105]. Although SZ95 sebocytes were found to express adiponectin at the mRNA level under normal conditions—which was down-regulated by a TLR1/2 activator—its secretion was not detectable in the supernatants, calling for further studies to reveal the possible biological role for sebocyte derived adiponectin [101]. Omentin-1 was also found to be present in normal SGs by immunohistochemical analysis; however, its role in the SG biology was not examined in detail [85].

## 3. Adipokines in Physiological Processes in the Skin

### 3.1. Melanogenesis

Melanogenesis is a complex process under the regulation of signals from the endocrine, immune as well as the nervous system, leading to the production of the pigment melanin in the melanosomes of melanocytes [106]. Interestingly, studies have shown that certain adipokines are also able to influence directly or indirectly the process of melanogenesis (Figure 1).

In the hypothesis that obesity might be associated with the reduction of melanogenesis, leptin became a major candidate, by increasing the levels of melanocyte stimulating hormone (MSH) antagonists in the circulation, that may result in a lower activity of circulating MSH and melanocortin 1 receptor (MC1R) [107].

Adiponectin receptors were found to be expressed also in immortalized human and mouse melanocytes. In response to adiponectin, melanocytes decreased the production of melanin and expressed melanogenesis-associated genes at a lower levels via the AMPK/CREB-regulated transcription co-activators (CRTCs) pathway, suggesting that adiponectin is an anti-melanogenic factor [108]. As a biological relevance, in hyperpigmentary disorders such as melasma, the mRNA levels of adiponectin and its receptors were also down-regulated in the basal layer of the lesional skin of melasma patients compared to the non-lesional skin [108,109]. However, the serum levels of adiponectin, just as of leptin, were not significantly different between melasma and control patients [108]. 

ZAG was also found to act on both normal and malignant melanocytes by inhibiting the production of melanin through post-transcriptional effects on tyrosinase protein [110] and inhibiting the proliferation of melanocytes, which may even have therapeutic implications in diseases with hypopigmentation such as vitiligo [111,112].

### 3.2. Hair Growth

Both unwanted loss (i.e., various alopecia forms) as well as undesired overgrowth (i.e., hirsutism and hypertrichosis) of the hair can substantially impair the quality of life of patients. Hair growth disorders are usually caused by a disturbance of the strictly regulated life cycle of the HFs, i.e., the hair cycle [113]. This life-long cycling consists of growth (“anagen”), regressive (“catagen”), and quasi-quiescent (“telogen”) phases [114]. Obviously, both the hair cycle and the hair growth are under tight and quite complex neuroendocrine control [115,116,117], and, of great importance, adipokines were also shown to contribute to this regulation.

Indeed, by using immunohistochemistry, leptin expression was demonstrated in the hair matrix, in the inner root sheath, as well as in the dermal papilla (DP) fibroblasts of human HFs, while its receptor (long isoform) was only found to be expressed by DP fibroblasts [118]. Pro-inflammatory cytokines as well as the growth factors that are known to suppress hair growth significantly reduced the production of leptin, whereas those that support hair growth did not affect its production, highlighting the possibility that leptinergic signaling may be involved in the initiation and/or maintenance of the anagen phase of HFs [118]. In line with this concept, transition from the first telogen to the second postnatal anagen phase was found to be delayed in db/db (leptin receptor-deficient) mice. Moreover, via JAK2, STAT3 and ERK1 signaling, the local injection of recombinant leptin promoted hair regrowth after shaving, by stimulating telogen-to-anagen transition [119]. Careful analysis of the hair cycle-dependent expression of leptin revealed that it was expressed in DP fibroblasts and follicular epithelial cells of catagen and telogen HFs. Intriguingly, in early anagen, leptin expression could only be detected in DP fibroblasts, and no leptin expression was found in late anagen HFs of wild-type mice [119]. In line with these observations, leptin expression in human DP fibroblasts was stronger in the catagen than in early anagen phase, and chemically mimicked hypoxia also promoted leptin release of DP fibroblasts isolated from male scalp HFs [119]. Based on these findings, the authors speculated that the hypoxic stress that usually accompanies catagen and telogen might be one of the positive regulators of the leptin production of DP fibroblasts, and this hypoxia-driven local leptin release may later play a role in initiating telogen-to-anagen transition. Intriguingly, however, although administration of leptin increased the number of nestin+ progenitor cells via the STAT3 pathway in the mesenchyme of organ-cultured human skin, it failed to do so in the HF epithelium [120], indicating that leptin might be a necessary—but not sufficient—player in the ignition of the telogen-to-anagen transition. Considering that leptin mRNA expression in the dermal white adipose tissue (DWAT) of mice was the lowest in the early anagen, increased in the late anagen, and peaked in the telogen phase [121], one might speculate that DWAT-derived leptin may be involved in driving telogen-to-anagen transition.

Intriguingly, certain data point to the fascinating possibility that putative leptinergic communication between DWAT and HFs may even be bidirectional. Indeed, it has recently been shown that DWAT undergoes major morphological changes during the spontaneous and induced murine HF cycling. Specifically, the thickness of DWAT is the greatest in late anagen, and decreases in the catagen and telogen phases [122]. Considering that leptin is thought to suppress lipid accumulation of DWAT [123] and that, as mentioned above, follicular leptin production is high in the catagen, telogen, and early anagen, but low in the late anagen phase [119], one might speculate that HF-derived local leptin production may reduce lipid accumulation of dermal white adipocytes.

Intriguingly, although the above findings introduce leptin as a potent pro-anagen agent, certain data argue that the in vivo regulation might be more complex. Indeed, higher plasma leptin concentration was found to be associated with a higher risk of androgenetic alopecia (AGA) in men, and plasma leptin level was significantly higher in subjects with advanced AGA compared to subjects with mild AGA [124], suggesting that disturbed leptinergic signaling may play a role in the pathogenesis of the said disease. Moreover, leptin was found to suppress the growth of organ-cultured mouse vibrissa [121], signifying that the final effect of leptin on hair growth (i.e., promotion or suppression) may also depend on the anatomical location as well as on yet unknown other biological variables.

Importantly, leptin is not the sole hair-relevant adipokine. Indeed, adiponectin receptors were shown to be expressed in human HFs [79]. Moreover, adiponectin treatment promoted hair shaft elongation in microdissected human HFs ex vivo [79]. Similarly, SERPINE1 was also found to be a hair growth promoting adipokine. Indeed, it was shown to be expressed in HFs of female BALB/c mice [52]. Importantly, SERPINE1–/– mice were found to exhibit delayed hair growth as compared to wild-type littermates [125], highlighting the possibility that SERPINE1 may indeed be a potent auto- and paracrine pro-anagen regulator.

In contrast to adiponectin and SERPINE1, overexpression of progranulin in keratin 5 (K5)+ cells decreased hair growth as well as the diameter of the hair shaft in mice by activating the p38 MAPK pathway [126]. Although only scant data are available in the literature, the serum level of the pro-inflammatory visfatin was not significantly different in alopecia areata (AA) patients and healthy individuals, according to a case control study [127]. In contrast, serum level of LCN2 was found to be elevated in hidradenitis suppurativa (HS) patients and LCN2 was also found to be up-regulated in lesional skin of HS patients [47,128]. Intriguingly, follicular LCN2 expression is restricted to the inner root sheath, and it is thought to be a marker for dysregulated keratinocyte differentiation in the interfollicular epidermis, because it was not present in healthy human epidermis, but it became prominent in parakeratotic lesions (e.g., in psoriasis) [46].

### 3.3. Wound Healing

Based on experiments in leptin-deficient animal models, numerous studies have confirmed that as an autocrine/paracrine regulator, leptin has a role also in regulating wound healing. During the normal healing process, leptin was found to be expressed at the wound site. Furthermore, immunohistochemical staining revealed that Ob-Rb was strongly expressed in the highly proliferative keratinocytes at the wound margin [129]. Moreover, by activation of the STAT3 pathway, topically administered leptin promoted wound healing in mouse skin by accelerating keratinocyte function and enhancing angiogenesis around the wounded area [130,131], while injection of anti-leptin antibodies into skin wounds reduced fibroblast functions. These findings altogether suggested that leptin from the circulation as well as produced by keratinocytes and fibroblasts might be an important regulatory factor in wound healing [132]. This observation was further supported by fibroblasts cultured from db/db mice, which exhibited impaired cell migration, vascular endothelial growth factor (VEGF) production and a disrupted response to hypoxia [133].

According to in vitro and in vivo experiments, visfatin may also effectively improve wound healing by enhancing the proliferation and migration of keratinocytes and fibroblasts via ERK1/2 and JNK1/2 signaling as well as via the induction of VEGF expression through the MAPK pathway [40].

LCN2 expression was also found to be elevated at the wound edge both in animal and in human experiments, suggesting that together with transcription factor 3 (Tcf3) and transcription factor 7-like 1 (Tcf7l1), LCN2 may regulate epidermal cell differentiation, migration and may accelerate wound healing via STAT3 signaling [134,135]. During the course of a scratch wound closure, an up-regulation of SERPINE1 via mitogen-activated extracellular signal-regulated kinase (MEK)/ERK and p38 signaling could be detected suggesting that the activation of this protein is required for epithelial cell migration and the global cutaneous injury response program [136,137]. Although elevated MCP-1 protein expression could be detected in normal wound repair, this protein is not derived from keratinocytes, but rather from infiltrating immune cells at the site of inflammation, with a role yet to be clarified [138].

Adiponectin in human and mouse fibroblasts plays an important role in hyaluronic acid metabolism and collagen synthesis, which may have beneficial effects also in wound healing [139]. Moreover, in adiponectin-deficient mice, keratinocyte proliferation and migration were impaired via the ERK signaling pathway during wound repair, underpinning that adiponectin is an important regulatory factor in this process [80].

Progranulin has also been identified as a wound-related growth factor in the human skin, because its mRNA expression was highly induced in dermal fibroblasts after injury and promoted the division and migration in an ERK- and PI3K-dependent manner [98].

## 4. Adipokines in Skin Diseases

### 4.1. Psoriasis

In psoriasis, the dermal inflammation induces the hyperproliferation of keratinocytes, which leads to the appearance of its hallmark plaques on predilection sites such as elbows, knees, lower back and scalp but can be present throughout the whole body. Importantly, a growing body of evidence supports the concept that psoriasis is not limited to the skin, but that it is rather a systemic inflammation, linked to metabolic diseases [140,141,142]. Several studies are available examining the relationship between psoriasis and the plasma or the tissue expression levels of different adipokines, with no clear conclusions which may be explained with methodological differences or incomparable study populations regarding the severity of psoriasis and/or comorbidities of the patients.

While studies showed that the serum leptin levels were elevated in psoriatic patients with normal body mass index (BMI) and correlated with the psoriasis area and severity index (PASI) scores [143,144,145]—suggesting that the increased leptin concentrations in the serum might not only be derived from adipocytes but also from dermal cells—another study did not find a relationship between the circulating leptin concentrations and the PASI score [146]. Making the picture more complicated, another study showed that the levels of leptin were decreased in psoriasis [147]. Regarding histological studies, leptin and leptin receptor expression were significantly higher in the skin of severe psoriasis patients with normal BMI than in patients with mild-moderate psoriasis and controls [143]. However, in another study that found correlation between the BMI and leptin serum levels but not with the disease severity in obese psoriatic patients, there were no differences in the expression levels of leptin in the skin between the examined groups. Moreover, the epidermal layer of lesional psoriatic skin exhibited lower leptin receptor expression than the normal or the uninvolved skin [148]. Interestingly, leptin mRNA expression in the subcutaneous adipose tissue positively correlated with circulating levels of leptin, as well as with the severity of psoriasis and the BMI in obese psoriatic patients, which highlights that the subcutaneous adipose tissue (and its adipokines) may play an important regulatory role in the pathogenesis psoriasis [149].

Elevated levels of resistin were consistently observed in the circulation of psoriasis patients and positively correlated with the PASI score [145,148,150]. The serum level of progranulin was also increased in patients with psoriasis and its expression was elevated in the affected skin [151]. SERPINE1 expression was found to be higher in the biopsies of psoriatic patients’ skin, however the limited data allows only speculation on its role [152]. Although increased levels of visfatin were also observed in the psoriatic patients, it correlated with the BMI, but not with the disease severity [153,154]. Furthermore, a gene expression study showed that the expression of visfatin was increased in peripheral blood monomorphonuclear cells of patients with psoriasis [154]. Chemerin expression may also be associated with the psoriasis progression, because its lowest levels were observed in chronic plaque psoriatic skin [155]. While the circulating RBP levels were in the normal range both in uninvolved and lesional psoriatic skin, studies with fetuin-A are more contradictory; indeed, one study found elevated serum fetuin-A levels in psoriasis whereas others did not [156,157,158,159]. Unfortunately, only limited number of studies are available on ZAG, with one showing that its immunoreactivity exhibited higher intensity in the epidermal cell layers of psoriatic patients which might be responsible for the process of desquamation [73].

Regarding anti-inflammatory adipokines, serum adiponectin and omentin levels were significantly lower in psoriatic patients compared to healthy controls, and studies found a negative correlation with disease severity [147,150,160,161,162,163,164]. Notably, both adipokines showed lower mRNA and protein expressions in the skin of patients with psoriasis [85,165]. Interestingly, topical or systemic anti-psoriatic treatment was shown to increase adiponectin and omentin levels in the psoriatic patient group [150,161,166] suggesting that both adipokines may play a protective role in the development of psoriasis. Altered vaspin expression might also contribute to the maintenance of psoriasis, because its epidermal expression was also down-regulated in human lesional psoriatic skin [86,87]; however, studies reported both an unaltered as well as significantly lower serum vaspin concentrations in psoriatic patients [86,167].

The number and size of SGs are known to decrease in psoriasis [168]. Importantly, SGs were found to express leptin, resistin, visfatin and SERPINE1 in lesional skin samples of patients with psoriasis. This raises the possibility that alterations in the number amount of SGs as well as in their adipokine-release pattern might contribute to the pathogenesis of psoriasis. [101].

Single nucleotide polymorphisms (SNPs) are the most common type of genetic variations, which result from a substitution of a single nucleotide in a specific position of the genome. SNPs in pro- and anti-inflammatory adipokine genes encoding leptin, leptin receptor, adiponectin and omentin were addressed in psoriasis; however, (with the exception of one study of the rs7799039 polymorphism in the leptin gene), researchers found no correlation or increased risk of psoriasis vulgaris with these polymorphisms [85,164,169,170,171].

**Figure 2:**
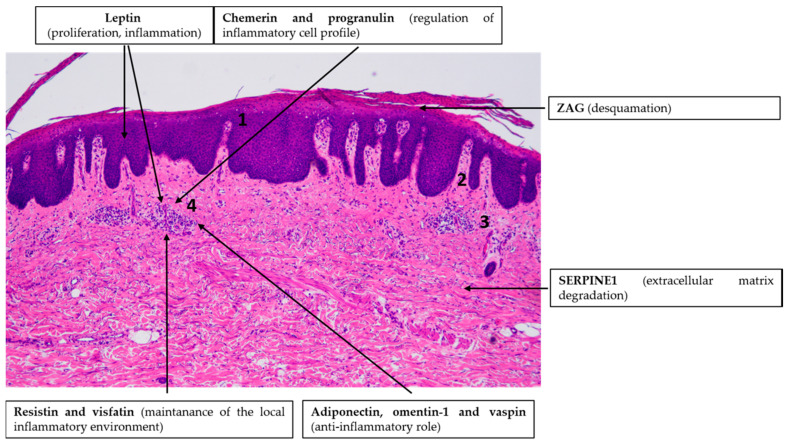
Adipokines might be involved in the pathogenesis of psoriasis vulgaris. Note the simplified characteristic histopathological findings in psoriasis skin: hyperproliferation of keratinocytes (1), elongation of the dermal papillae (2), dilated blood vessels (3) and immune cell infiltration in the dermis (4). Hematoxylin eosin staining; original magnification: 50×.

The putative role of adipokine signaling in the pathogenesis of psoriasis is overviewed in Figure 2.

### 4.2. Atopic Dermatitis (AD)

AD is a common chronic inflammatory skin disease usually with an early onset. People with AD have a dry and itchy skin with inflammation [172].

Although there is a consensus that adipokines might have a potential role in the pathogenesis of AD, further prospective, controlled studies with larger cohorts of patients are needed to confirm whether the alterations in adipokine levels precedes or “only” accompanies the manifestation of AD.

Regarding leptin, studies showed that its serum levels were significantly elevated in children and adult patients with IgE-associated AD compared to healthy controls [173,174], whereas others found that the circulating leptin levels were decreased or had no statistically significant association in patients with AD [175,176,177].

In the case of resistin and LCN2 similar controversial observations are available. While elevated serum levels of resistin were reported in children with AD compared to healthy controls [178], studies with opposing results are also available [174,179]. Serum LCN2 levels were also significantly higher in patients with AD [180]; however, other studies reported its decreased concentrations [174,181].

ZAG, which might have a role in modulating the skin barrier functions and immune responses through influencing the Notch signaling, exhibited a reduced expression in both the serum and the skin of AD patients [182].

A decreased blood level of adiponectin has been found in AD [174,175], whereas no significant differences were detected in its serum levels when correlated with the SCORAD severity index score. Interestingly, the mean adiponectin levels in extrinsic AD were lower than in intrinsic AD [183].

In genetic studies, significant associations have been detected between the rs2167270 polymorphism of leptin gene (A19G) and the rs3745367 polymorphism of resistin gene (G > A) with the incidence and development of AD [184,185].

**Figure 3:**
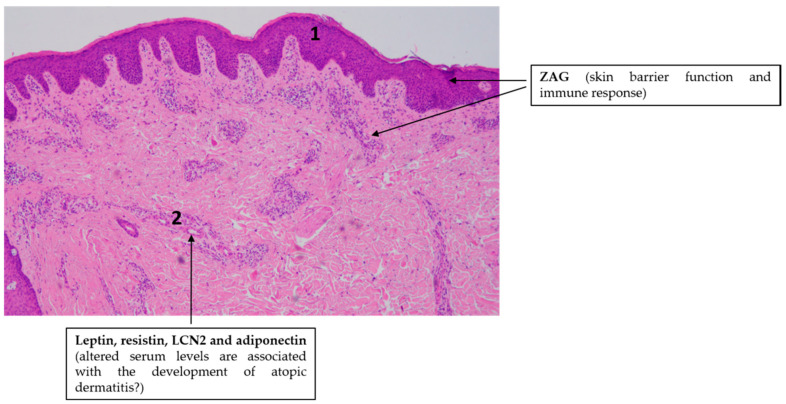
Adipokines might be involved in the pathogenesis of atopic dermatitis. Note the simplified characteristic histopathological findings in AD skin: slight epidermal hyperplasia with impaired barrier function (1) and immune cell infiltration in the dermis (2). Hematoxylin eosin staining; original magnification: 50×.

The putative role of adipokine signaling in the pathogenesis of AD is overviewed in Figure 3.

### 4.3. Acne Vulgaris

Acne vulgaris is one of the most common inflammatory skin diseases, involving the pilosebaceous unit in SG rich areas of the body such as the face, the chest and the back [99]. Considering that sebocytes, similarly to adipocytes, are highly active in lipid metabolism, we previously performed a comprehensive analysis and revealed that SGs express a characteristic set of adipokines also in acne skin samples, and their secretion may be modulated by acne related pathogenic (TLR) and therapeutic (isotretinoin) stimuli. [101].

Among the adipokines, the effects of leptin has been studied the most. Acting in an autocrine manner, leptin may be involved in an increased lipid metabolism and pro-inflammatory cytokine/enzyme production by activating the STAT3 pathway as observed in human SZ95 sebocytes in vitro, which resembled the changes detected in the sebum of acne patients [102,186]. While through augmenting Th17/IL-17 signaling and enhancing the secretion of pro-inflammatory cytokines by immune cells, leptin may also be involved in generating the pathological immune-milieu characteristic for lesional acne skin [187,188,189,190]. Ductal hypoxia, occurring in the obstructed follicles, has been also linked to the development of acne and hypoxia-inducible factor 1-α (HIF-1α) was shown to promote leptin expression [91,191]. Because mTORC1 is a potential regulator of HIF-1α, activation of the mTORC1 pathway therefore could also alter the sebocyte-derived leptin production [104]. Interestingly, the findings that leptin and other factors from the “Western diet” may activate the mTORC1 pathway, which, in turn, further enhances leptin production, suggest a possible role for the leptin-mTORC1 axis to link nutrition with the development of acne [104,192].

Although linking the dysregulated adipokine function with acne vulgaris is an intriguing area, the available data on the serum levels can only be evaluated to a limited extent. The results are inconsistent and difficult to interpret or to compare also in the context of acne because: (1) small groups of patients were studied; (2) there are mostly insufficient data regarding acne severity; and (3) significant heterogeneity can be observed among the studies.

Although some papers demonstrated that there was no significant difference between circulating leptin, adiponectin, and RBP4 levels in patients with acne vulgaris, others found higher leptin and lower adiponectin serum concentrations [193,194,195,196]. Interestingly, isotretinoin (the most effective anti-acne agent) altered adiponectin levels and decreased leptin levels, which were significantly lower in the acne patients compared with the control group already at the basal levels [196,197].

In the available genetic studies, the resistin gene rs1862513 (+299G > A) and rs3745367 (−420C > G) polymorphisms were strongly associated with familial acne vulgaris and the severity of symptoms but not with the acne types [198,199]. Moreover, the omentin gene rs2274907 (Val109/Val109) polymorphism leading to protein dysfunction, increases the predisposition to acne vulgaris [200].

**Figure 4:**
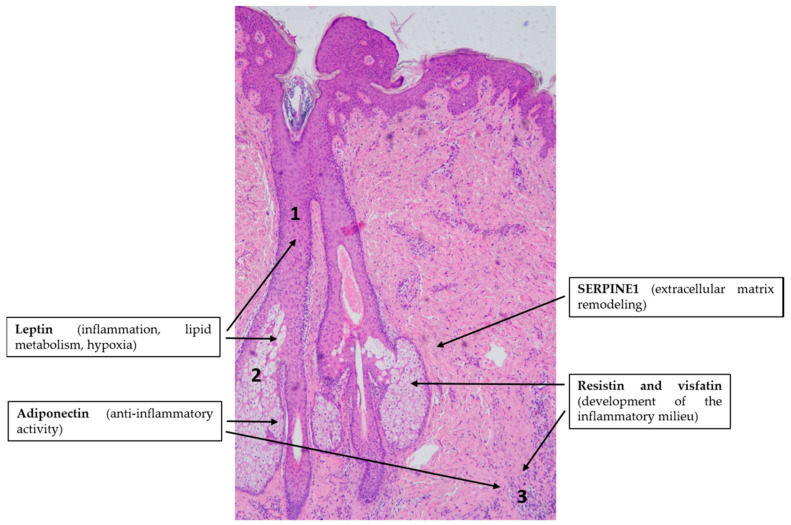
Adipokines might be involved in the pathogenesis of acne vulgaris. Note the simplified characteristic histopathological findings in acne: ductal hyperkeratosis (1), altered sebum production by sebocytes (2) and immune cell infiltration in the dermis (3). Hematoxylin eosin staining; original magnification: 50×.

The putative role of adipokine signaling in the pathogenesis of acne is overviewed in Figure 4.

### 4.4. Rosacea

Rosacea is a chronic inflammatory skin disease with clinical features such as dry skin, teleangiectasias and papulopustular lesions primarily on the face, but it may have ocular manifestations as well. In its development, genetic and environmental factors such as colonization of Demodex folliculorum (D. folliculorum) are suggested to play roles [201]. Although pro-inflammatory cytokines and chemokines were addressed [202,203,204], little is known about how adipokines may be involved in the development and progression of rosacea. In an in vitro rosacea skin lesion model, in response to the antibacterial peptide LL-37 (cathelicidin) the production of MCP-1 was increased in HaCaT keratinocytes through the activation of JAK2/STAT3 signaling, suggesting that these pathways may play an important role in the inflammation caused by D. folliculorum [205]. Furthermore, in ex vivo skin models, as well as in NHK and HaCaT cells, anti-parasitic and anti-inflammatory agents (e.g., ivermectin or artemisinin) reduced the LL-37-induced activation of NF-κB signaling and the secretion of MCP-1 [206,207]. However, in a prospective, observational study, there were no differences in serum and tear MCP-1 levels between the rosacea patients with or without ocular involvement and the control groups [208]. As SERPINs are known to interact with kallikreins, which are important regulators of the immune barrier alteration and skin desquamation, they might be involved in modifying the physiological structures of the epidermis, which can lead to the typical clinical signs in rosacea [209,210,211]. Epidermal LCN2 levels were also significantly higher in papulopustular rosacea compared with controls and this expression level was strongly associated with the levels of permeability barrier structural components [212]. Leptin, adiponectin, resistin, SERPINE1 and visfatin were also detectable in the SGs of rosacea-involved skin, suggesting that through the secretion of these adipokines, SGs may contribute to the development of the characteristic inflammatory milieu in rosacea [101].

**Figure 5:**
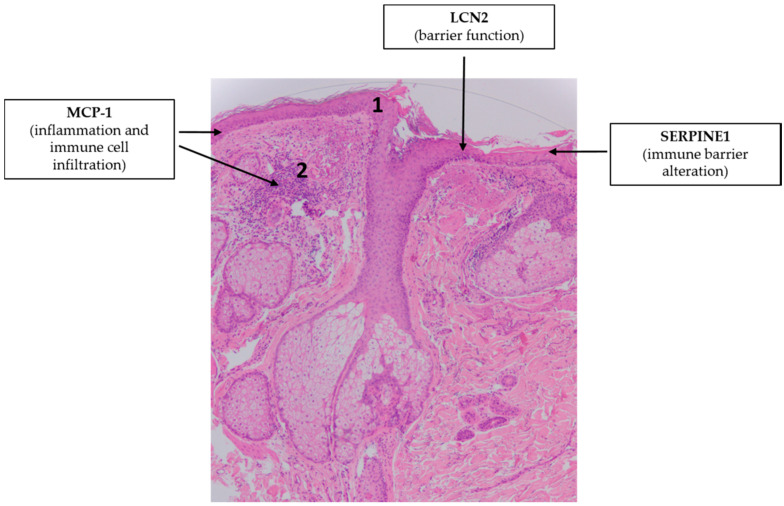
Adipokines may be involved in the pathogenesis of rosacea. Note the simplified characteristic histopathological findings in rosacea: impaired barrier function (1), interfollicular inflammation in the dermis (2). Hematoxylin eosin staining; original magnification: 50×.

The putative role of adipokine signaling in the pathogenesis of rosacea is overviewed in Figure 5.

### 4.5. Malignant Melanoma

Melanoma is the most aggressive skin tumor. Its risk factors include (among others) genetic background, sun exposure, and skin type [213].

Although, a growing body of evidence suggests that obesity is also a risk factor for human melanoma incidence, the available data are controversial. Some studies found a positive association between obesity and the risk of melanoma, while others have not [214,215,216]. Thus, elucidation of the putative role of adipokines both from systemic as well as from local origin requires further studies.

Based on experiments performed by using genetically obese mice as well as mice suffering from diet-induced obesity, researchers found that adipokines might enhance the proliferation of melanoma cells and thus obesity could be considered as a tumor-promoting factor, whose presence may result in a poor prognosis of melanoma [217,218,219]. Indeed, melanoma progression was found to be partially decreased when the body weight was controlled by anti-obesity drugs or restriction of the calorie intake [219].

Leptin and its functional receptor was detected in human melanoma cell lines as well as in histological sections in melanomas. Moreover, the expression of Ob-Rb was increased in the WM793 and WM35 melanoma cell lines compared to normal melanocytes [220,221]. In line with this data, serum leptin levels correlated positively with melanoma risk; however, a reverse correlation was found in the case of tumor thickness and serum levels of leptin receptor [222,223]. In human WM793 and WM35 melanoma cell lines, leptin treatment activated the MAPK pathway and enhanced cell proliferation, suggesting that leptin may act as a melanoma growth factor, and may contribute to the uncontrolled proliferation of these cells [220]. Leptin treatment following the subcutaneous injection of melanoma cells into C57BL/6 mice resulted in larger tumor size, higher capillary density and increased plasma levels of VEGF, supporting that, as an angiogenic and mitogenic factor, leptin could promote melanoma growth [224]. Resistin, apelin and visfatin are also able to act on melanoma cells by enhancing the proliferation, accelerating the tumor growth and triggering the redox adaptation responses through an insulin receptor independent pathway [217,225,226]. Moreover, human MeWo and HMCB melanoma cells are able to produce visfatin, which can activate the MAPK, the AKT and the NF-κB pathways [227]. Providing further relevance for visfatin in the progression of melanoma, its elevated levels were found in the serum of patients with BRAF-mutated metastatic melanoma with decreasing concentrations in response to BRAF/MEK inhibitor therapy [228]. The decreased expression of chemerin in murine and human melanomas was associated with a poor prognosis, suggesting an impaired anti-tumor NK cell recruitment in the background, which can be observed in the tumor microenvironment, aggravating the disease [229,230]. SERPINE1 may be involved in the regulation of the invasion of malignant cells and the formation of metastasis with its effect on tissue matrix remodeling; thus, it may be considered as a poor prognostic biomarker in human melanomas [231].

The protective role of adiponectin in melanoma pathogenesis also calls for further investigations, as a remarkable but statistically non-significant reverse correlation of serum adiponectin levels were found in melanoma patients in a case-control study [232].

**Figure 6:**
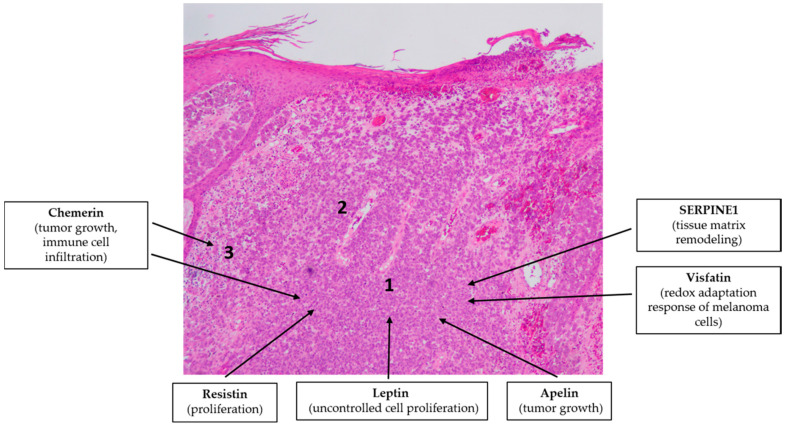
Adipokines might be involved in the pathogenesis of malignant melanoma. Note the simplified characteristic histopathological findings in melanoma: invasion of the malignant cells (1), increased vascularization (2) and immune cell infiltration (3). Hematoxylin eosin staining; original magnification: 50×.

The putative role of adipokine signaling in the pathogenesis of melanoma is overviewed in Figure 6.

## 5. Conclusions

In this review, we attempted to summarize and highlight the complexity of the adipokines and their related signaling pathways in the skin, which may lead to proven and in some cases presumed changes in the function of various skin cell types. While adipokines, both as targets and tools, are potential candidates for therapies not only in obesity and metabolic diseases, but also in lipodystrophy, infertility and bone growth [233], there is still limited data on their possible application to treat skin diseases. Moreover, we need not only to understand their complex actions in the skin and with that the possible side effects, but their targeted delivery to the skin cells just as utilizing the mechanisms that could induce or inhibit their local expression/secretion should be revealed as well. In summary, it appears that our knowledge on the role of adipokines to regulate the (patho)physiological changes in the skin is still in an early phase, calling for further studies to reveal their biological relevance and to deliver adipokine-based therapeutic solutions.

## Abbreviations

5-LOX	5-lipoxygenase
AA	Alopecia areata
ACRP/AdipoQ	Adiponectin
AD	Atopic dermatitis
ADIPOR	Adiponectin receptor
AGA	Androgenetic alopecia
Akt	Protein kinase B
AMPK	AMP-activated protein kinase
APJ	Apelin receptor
BMI	Body mass index
BRAF	B-Raf proto-oncogene serine/threonine kinase
C/EBP	CCAAT/enhancer-binding protein
CCL2	C-C motif ligand 2
CMKLR1	Chemerin chemokine-like receptor 1
COX-2	Cyclooxygenase-2
CRTCs	CREB-regulated transcription co-activators
CXCL	(C-X-C motif) ligand
DC	Dendritic cell
DGAT	Diglyceride acyltransferase
DP	Dermal papilla
DMBPA/TPA	Dimethylbenz[a]anthracene/12-O tetradecanoylphorbol-13-acetate
DWAT	Dermal white adipose tissue
EGF	Epidermal growth factor
EGFR	Epidermal growth factor receptor
ERK	Extracellular signal-regulated kinases
GPR1	G protein-coupled receptor 1
hBD-2	Human β-defensin-2
HF	Hair follicle
HIF-1α	Hypoxia-inducible factor 1-α
HS	Hidradenitis suppurativa
IFN-γ	Interferon-γ
IL	Interleukin
JAK2	Janus kinase 2
JNK	c-Jun N-terminal kinase
LCN2	Lipocalin2
LPS	Lipopolysaccharide
MAPK	Mitogen-activated protein kinase
MC1R	Melanocortin 1 receptor
MCP-1	Monocyte chemoattractant protein-1
MEK	Mitogen-activated protein kinase kinase
MMP	Matrix metalloproteinase
MSH	Melanocyte stimulating hormone
mTORC1	Mammalian target of rapamycin complex 1
NAMPT	Nicotinamide phosphoribosyltransferase
NF-κB	Nuclear factor kappa-β
NGAL	Neutrophil gelatinase–associated lipocalin
NHK	Normal human keratinocyte
NK cell	Natural killer cell
Ob-Rb	Leptin receptor
PAI-1	Plasminogen activator inhibitor type-1
PASI	Psoriasis area and severity index
PBEF	Pre-B-cell colony-enhancing factor
PI3K	Phosphatidylinositol 3-kinas
RARRES2	Retinoic acid receptor responder 2
RBP	Retinol-binding protein
ROS	Reactive oxygen species
SCORAD	SCORing Atopic Dermatitis
SERPIN	Serine protease inhibitor
SG	Sebaceous gland
SNP	Single nucleotide polymorphism
SOCS-3	Suppressor of cytokine signaling 3
SREBP1	Sterol regulatory element binding protein 1
STAT3	Signal transducer and activator of transcription factor 3
STRA6	Signaling receptor and transporter of retinol STRA6
SZ95	Human sebaceous gland cell line
Tcf	Transcription factor
TGFβ	Transforming growth factor β
Th	T helper cell
TIG2	Tazarotene-induced gene 2 protein
TLR	Toll-like receptor
TNF-α	Tumor necrosis factor-α
UVB	Ultraviolet B
VEGF	Vascular endothelial growth factor;
WAT	White adipose tissue
ZAG	Zinc α2-Glycoprotein
